# Inhibition of Soluble Epoxide Hydrolase Ameliorates Phenotype and Cognitive Abilities in a Murine Model of Niemann Pick Type C Disease

**DOI:** 10.3390/ijms22073409

**Published:** 2021-03-26

**Authors:** Christian Griñán-Ferré, Júlia Companys-Alemany, Júlia Jarné-Ferrer, Sandra Codony, Celia González-Castillo, Daniel Ortuño-Sahagún, Lluïsa Vilageliu, Daniel Grinberg, Santiago Vázquez, Mercè Pallàs

**Affiliations:** 1 Pharmacology and Toxicology Section and Institute of Neuroscience, Faculty of Pharmacy and Food Sciences, University of Barcelona, Av. Joan XXIII, 27-31, 08028 Barcelona, Spain; christian.grinan@ub.edu (C.G.-F.); juliacompanysalemany@gmail.com (J.C.-A.); jjarnefe18@alumnes.ub.edu (J.J.-F.); 2 Laboratory of Medicinal Chemistry (CSIC, Associated Unit), Faculty of Pharmacy and Food Sciences and Institute of Biomedicine (IBUB), University of Barcelona, Av. Joan XXIII, 27-31, 08028 Barcelona, Spain; sandra.codony@ub.edu (S.C.); svazquez@ub.edu (S.V.); 3 Tecnológico de Monterrey, Escuela de Medicina y Ciencias de la Salud, Campus Guadalajara, Zapopan, 45201 Jalisco, Mexico; celia_glz@tec.mex; 4 Laboratorio de Neuroinmunobiología Molecular, Instituto de Investigación en Ciencias Biomédicas (IICB), Centro Universitario de Ciencias de la Salud (CUCS), Universidad de Guadalajara, Jalisco 44340, Mexico; daniel.ortuno@academicos.udg.mx; 5 Department of Genetics, Microbiology and Statistics, Faculty of Biology, University of Barcelona, 08028 Barcelona, Spain; lvilageliu@ub.edu (L.V.); dgrinberg@ub.edu (D.G.); 6 Institut de Biomedicina de la UB (IBUB)-Institut de Recerca Sant Joan de Déu (IRSJD), 08028 Barcelona, Spain; 7 Centre for Biomedical Research on Rare Diseases (CIBERER), 08028 Barcelona, Spain

**Keywords:** Niemann–Pick type C, soluble epoxide hydrolase, autophagy, cognitive decline, lifespan, inflammation, cholesterol, sphingolipids

## Abstract

Niemann–Pick type C (NPC) disease is a rare autosomal recessive inherited childhood neurodegenerative disease characterized by the accumulation of cholesterol and glycosphingolipids, involving the autophagy-lysosome system. Inhibition of soluble epoxide hydrolase (sEH), an enzyme that metabolizes epoxy fatty acids (EpFAs) to 12-diols, exerts beneficial effects in modulating inflammation and autophagy, critical features of the NPC disease. This study aims to evaluate the effects of UB-EV-52, an sEH inhibitor (sEHi), in an NPC mouse model (Npc) by administering it for 4 weeks (5 mg/kg/day). Behavioral and cognitive tests (open-field test (OF)), elevated plus maze (EPM), novel object recognition test (NORT) and object location test (OLT) demonstrated that the treatment produced an improvement in short- and long-term memory as well as in spatial memory. Furthermore, UB-EV-52 treatment increased body weight and lifespan by 25% and reduced gene expression of the inflammatory markers (i.e., *Il-1β* and *Mcp1*) and enhanced oxidative stress (OS) markers (*iNOS and Hmox1*) in the treated Npc mice group. As for autophagic markers, surprisingly, we found significantly reduced levels of LC3B-II/LC3B-I ratio and significantly reduced brain protein levels of lysosomal-associated membrane protein-1 (LAMP-1) in treated Npc mice group compared to untreated ones in hippocampal tissue. Lipid profile analysis showed a significant reduction of lipid storage in the liver and some slight changes in homogenated brain tissue in the treated NPC mice compared to the untreated groups. Therefore, our results suggest that pharmacological inhibition of sEH ameliorates most of the characteristic features of NPC mice, demonstrating that sEH can be considered a potential therapeutic target for this disease.

## 1. Introduction

Niemann–Pick disease type C (NPC, MIM # 257220) is a rare autosomal recessive neurodegenerative disease (1/120,000 live births in Europe). The disorder is characterized by a defect in lipid trafficking that results in an inability to process cellular cholesterol, accompanied by a secondary accumulation of glycosphingolipids in the lysosomes of affected individuals [1,2]. It is caused by mutations in the NPC1 gene (this occurs in 95% of diagnosed cases) or in the NPC2 gene [3]. NPC1 is a late-endosomal transmembrane protein, which binds cholesterol, whereas NPC2 resides in the lysosomal lumen and transfers cholesterol to NPC1 [4]. Therefore, defects in NPC1 or NPC2 proteins lead to the accumulation of cholesterol and glycosphingolipids in lysosomes and cause hepatic, pulmonary and neuropsychiatric disorders in humans [4,5,6]. The first clinical manifestations of NPC appear during childhood and are usually diagnosed before 10 years of age. Patients often present with cerebellar ataxia, progressive behavioral and cognitive disabilities, as well as dementia [5]. Adult manifestation (15 years and older) is rare, progression is usually much slower, and patients present with a broad phenotypic spectrum similar to childhood manifestation, including epilepsy and parkinsonism syndrome. In addition, disease progression and life expectancy are causally related to the occurrence of neurological symptoms [5]. Cellular and molecular hallmarks in the central nervous system (CNS) are the presence of lipids inclusions, changes in the composition of lipid content, increased cholesterol storage and multiple sphingolipids in the membranes of neurons [7]. These changes in the NPC brain are accompanied by mitochondrial dysfunction, oxidative stress (OS) and a strong inflammatory component (gliosis in grey and white matter, microglial activation) that ultimately lead to brain-wide synaptic disruption phenomena [4,8].

Moreover, protein dysregulation is also present in NPC tissues. Gene expression analysis of NPC patients has revealed molecular similarities with neurodegenerative diseases, such as accumulation of hyperphosphorylated tau in neurofibrillary tangles (NTFs) and abnormal processing of β-amyloid [9,10]. Cells have different efficient mechanisms to eliminate damaged organelles, aberrant protein aggregation and other debris cell functioning in normal or pathological situations. Autophagy is one such mechanism that involves a large control of lysosome-mediated functions [11]. The process of autophagy is necessary for maintaining cellular homeostasis and cell survival, especially in situations of amino acid starvation or cellular stress. In addition, autophagy is required for normal CNS development and function [12]. It has been implicated in most neurodegenerative diseases and other human diseases, such as cardiovascular diseases, immune disorders, or cancer [13]. It is well known that many factors affect the autophagic process in the brain, including inflammation, OS, alterations in synaptic plasticity, and aberrant accumulation of lipid metabolites. In NPC disease, induction of autophagy through increased vacuoles and accumulation of lipidated LC3 was reported [14].

Furthermore, it has been established that the class III-PI3K/beclin-1 complex is the key factor involved in autophagy induction in NPC1 deficiency because its expression is slightly elevated in NPC1-deficient mouse tissue and human fibroblasts. Moreover, knock-down of beclin-1 by siRNA decreases the degradation of long-lived protein [15]. Therefore, the etiopathogenesis of NPC disease is mainly associated with aberrant cholesterol accumulation and two alterations, mainly inflammation and autophagy.

To date, successful therapy for NPC disease has been elusive with modest results in motor and cognitive impairment by different strategies, including miglustat [16], hydroxypropyl beta-cyclodextrins (HPßCD) [17] or anti-inflammatory drugs [18]. Combination therapy with HPßCD, allopregnanolone and miglustat has been shown to delay disease onset and increase the lifespan of NPC1 mice by reducing intraneuronal lipid storage and positively influencing motor dysfunction [19]. The positive effect of miglustat monotherapy was further enhanced by additional dual therapy with curcumin and miglustat and triple combination therapy [20]. A modest but not negligible positive action of cyclodextrins and miglustat has been described in humans. Unfortunately, no specific and effective treatment is available at the moment. Therefore, identifying promising therapeutic and preventive strategies for this disease has so far been a challenge.

The enzyme soluble epoxide hydrolase (sEH; EC 3.3.2.10) is emerging as a pharmacological target, as its inhibition has been shown to have beneficial effects in metabolic disorders [21] and in neurodegenerative diseases, such as Alzheimer’s disease (AD) [22,23] and Parkinson’s disease (PD) [24]. In the arachidonic acid (AA) cascade, cytochrome P450 epoxygenases convert AA to epoxyeicosatrienoic acids (EETs), which are hydrolyzed by sEH into their corresponding dihydroxyicosatrienoic acids (DHETs) (DHETs) [25]. EETs are potent cell signaling molecules that regulate key events, such as ameliorating mitochondrial dysfunction [26], reducing apoptosis [27], modulating the autophagic response [28], and reducing inflammation [29]. In addition, EETs modulate specific processes in neuronal and glial cells, as well as the communication between different cell types [23]. Blockade of sEH reduces the deleterious effects of ischemic stroke and subarachnoid hemorrhage [30]. Furthermore, inhibition of sEH reduces cognitive impairment in AD mouse models by reducing OS, endoplasmic reticulum (ER) stress and, most importantly, by reducing mediators of neuroinflammation, such as tumor necrosis factor (TNF-α) and interleukin 1 beta (IL-1β) [22]. Finally, 14,15-EETs have recently been described to reduce cholesterol accumulation in human fibroblasts from NPC patients by reducing cholesterol synthesis and improving autophagic flux [31].

As mentioned above, despite the research conducted for a useful treatment for NPC disease, a successful therapeutic tool has not been identified. Therefore, an anti-inflammatory, antioxidant or more specific drug to improve the prognosis for NPC patients may be a new insight [8]. In the present study, we demonstrated that the use of sEH as a target to fight this devastating disease might be a new starting point for the development of therapies against NPC disease. To this end, we tested a well-characterized sEHi (UB-EV-52) in a mouse model of the disease [31], which can inhibit the sEH at the brain level through an in vivo thermal shift assay (CETSA) [22], demonstrating target engagement. Next, we focused our effort on the distinctive features of the disease, such as cognition, survival, changes in lipid accumulation, neuroinflammation, OS, synaptic plasticity, and activation of the autophagic process.

## 2. Results

### 2.1. Changes in Bodyweight and Survival after Treatment with UB-EV-52

**Figure 1:**
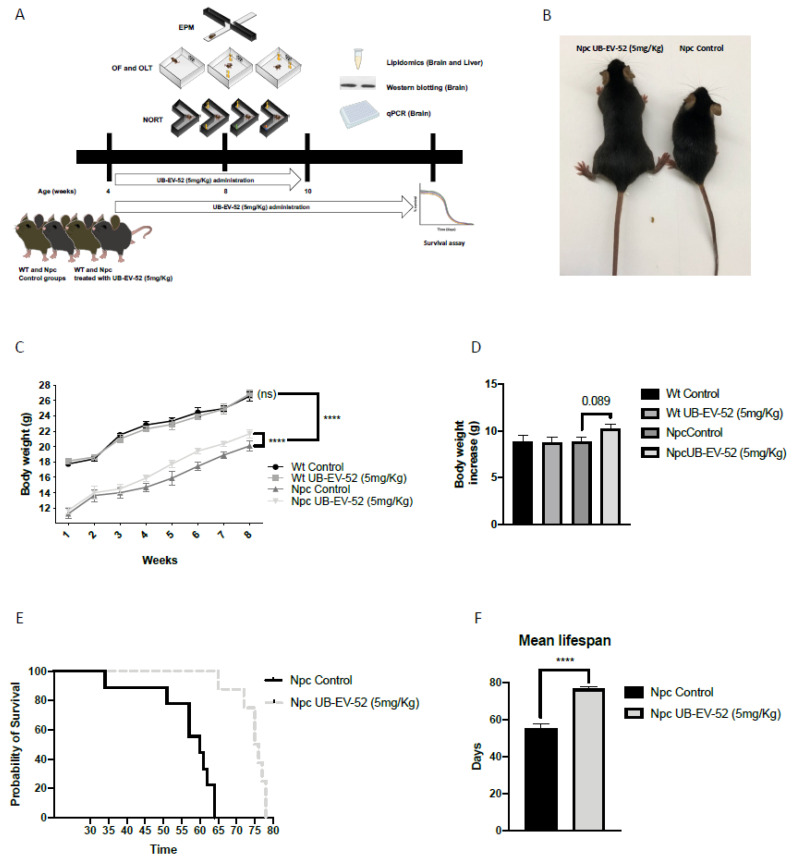
Schematic of experimental design (**A**), mouse phenotype (**B**), body weight curve results in females and males (**C**), total body weight gain results in females and males (**D**), survival curve in females and males (**E**), average lifespan in females and males (**F**). Values represented are mean ± standard error of the mean (SEM); *n* = 48 (wild-type (Wt) control *n* = 12, Wt UB-EV-52 (5 mg/kg) *n* = 12, Niemann–Pick type C (Npc) control *n* = 12, and Npc UB-EV-52 (5 mg/kg) = 12). **** *p* < 0.0001.

Bodyweight was measured weekly during the intervention. From baseline (1 week of age), Npc mice were significantly lower compared to wild-type (Wt) mice, whereas UB-EV-52 treatment significantly increased the bodyweight of Npc mice (Figure 1C). Furthermore, as expected, the treatment did not change the mean body weight gain of Wt animals, being 8.81 g for the Wt control group and 8.73 g for the Wt treated group (Figure 1D). In contrast, there was a clear trend toward an increase in the mean bodyweight of the Npc-treated animals (10.22 g) compared to the Npc control group (8.84 g) (Figure 1D).

Significantly, treatment with sEHi delayed mortality of Npc mice compared to untreated animals, as shown in the Kaplan–Meier survival curve presented in (Figure 1D). Accordingly, UB-EV-52 increased the lifespan of NPC by 25% (Figure 1E).

### 2.2. Sphingolipid and Cholesterol Profiles in Mouse Tissues and the Effect of UB-EV-52 Treatment

To evaluate the effect of sEH inhibition in Npc and Wt mice on lipid storage, a relevant phenotype that is observed in NPC patients, brain and liver sphingolipid levels—and cholesterol amounts were determined by gas chromatography–mass spectrometry.

As expected, significant differences were observed between Wt and Npc mice. Concretely, Npc animals showed a significant accumulation of sphingomyelin, dihydrosphingomyelin, ceramides and gangliosides (GM2 and GM3) (Supplementary Figures S1 and S2). Significant cholesterol accumulation was observed in the liver and brain of Npc mice, compared with the Wt group (Supplementary Figures S1C and S2C). However, the results showed that UB-EV-52 subtly modified the lipid storage profile in the brain of treated animals (Supplementary Figure S1). In the liver, a slight decrease in the lipid species evaluated was found (Supplementary Figure S2), which reached statistical significance only for dihydrosphingomyelin (Supplementary Figure S2C).

### 2.3. Improvement of Behavioral Performance after Treatment with UB-EV-25

**Figure 2:**
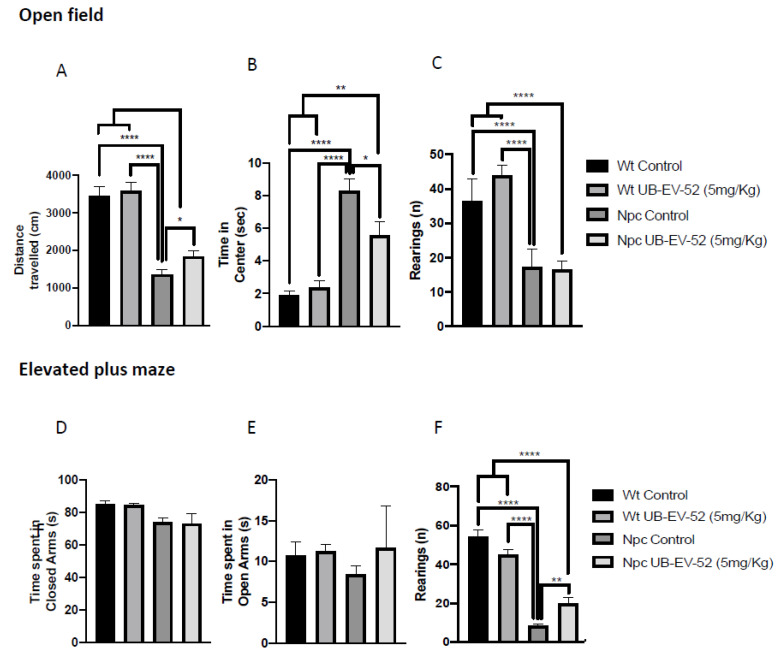
OFT results. Distance traveled in both females and males (**A**), time spent in the central zone (**B**), rearings (**C**). EPM results: time spent in open arms (**D**), time spent in closed arms (**E**), rearings (**F**). Values represented are mean ± standard error of the mean (SEM); *n* = 48 (Wt control *n* = 12, Wt UB-EV-52 (5 mg/kg) *n* = 12, Npc control *n* = 12, and Npc UB-EV-52 (5 mg/kg) = 12). * *p* < 0.05; ** *p* < 0.01; **** *p* < 0.0001.

Locomotor activity was analyzed after treatment with UB-EV-52 in the open field test (OFT) paradigm. The results obtained showed that Npc mice exhibited decreased locomotor activity, as evidenced by a significant decrease in distance traveled compared to the Wt group (Figure 2A). In addition, Npc mice stayed longer in the center of the arena compared to the Wt group. They exhibited a decrease in vertical activity, quantified by the number of rearings, indicating a disease-associated limitation in their activity (Figure 2C). UB-EV-52 did not modify locomotor activity in Wt mice, while it improved both parameters: distance traveled and time on center for Npc mice, confirming an important change in this characteristic feature of NPC disease (Figure 2A). Other parameters measured in OFT are presented in Table S1.

Regarding anxiety-like behavior, we studied several parameters using an elevated plus maze (EPM). The result showed no significant changes for either phenotype or treatment conditions compared to the time mice spent in open or closed arms at the age studied (Figure 2D,E). In contrast, lower vertical activity in this maze was observed for the Npc group compared to the Wt group, as well as a significant recovery in UB-EV-52-treated animals (Figure 2F). Other parameters measured in the EPM are presented in Table S2 (Supplementary Material).

### 2.4. Effect of UB-EV-52 Treatment on Cognitive Abilities of Npc Mice

**Figure 3:**
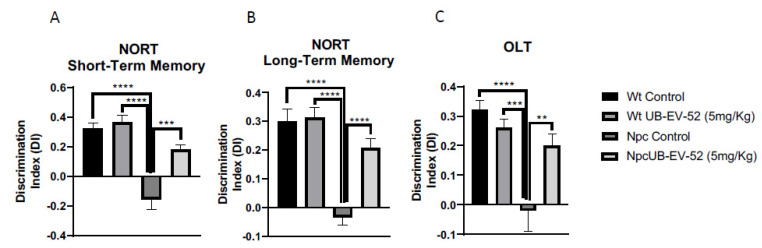
Novel object recognition test (NORT) results for short-term memory in both females and males (**A**), and long-term memory (**B**). Object location test (OLT) results (**C**). Values represented are mean ± standard error of the mean (SEM); *n* = 48 (Wt control *n* = 12, Wt UB-EV-52 (5 mg/kg) *n* = 12, Npc control *n* = 12, and Npc UB-EV-52 (5 mg/kg) = 12); discrimination index (DI). ** *p* < 0.01; *** *p* < 0.001; **** *p* < 0.0001.

The novel object recognition test (NORT) was used to assess cognitive performance after the UB-EV-52 treatment. This test has been previously used in the Npc mouse model to demonstrate cognitive impairment [31]. The NORT test was performed at 8 weeks of age, and analysis demonstrated that Npc showed a reduced discrimination index (DI) compared to age-matched Wt mice in the 2 h or 24 h test (Figure 3A,B). However, the UB-EV-52-treated Npc group exhibited significantly reduced cognitive deficits in short- and long-term memories determined for their Npc littermates. These results demonstrated beneficial effects on cognition following pharmacological inhibition of sEH, restoring it to a level similar to the Wt phenotype (Figure 3A,B).

Additionally, the object location test (OLT) paradigm was used to assess spatial memory. The results reinforced the NORT values and denoted a significant impairment of spatial memory in Npc compared to Wt mice. In addition, UB-EV-52-treated Npc mice exhibited longer exploration time for the location of a novel object, pointing out an improvement in spatial cognitive abilities after sEHi treatment (Figure 3C).

### 2.5. Reduction of Neuroinflammatory and Oxidative Stress Markers after UB-EV-52 Treatment in Npc Mice

**Figure 4:**
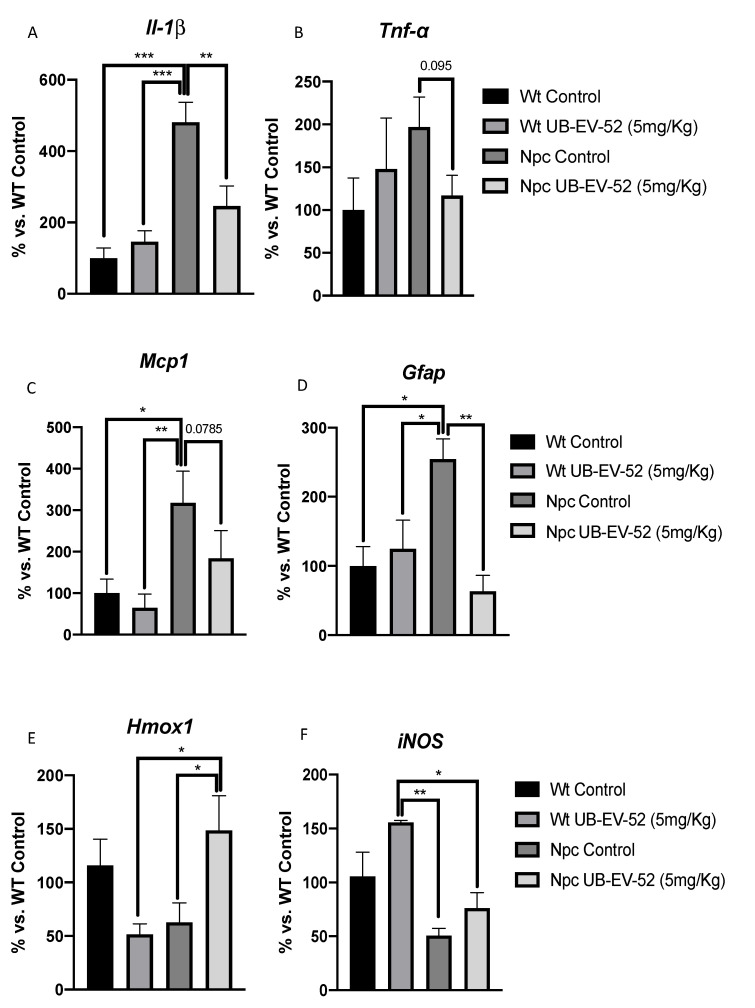
Gene expression of inflammatory markers *Il1-β* (**A**), *Tnf-α* (**B**), *Mcp1* (**C**), *Gfap* (**D**), *Hmox1* (**E**) and *iNOS* (**F**) from hippocampal tissue in females and males. Gene expression levels were determined by real-time PCR. Bar graphs values in are 100% adjusted for gene expression in the Wt control group. Values represented are mean ± standard error of the mean (SEM); *n* = 24 (Wt control *n* = 6, Wt UB-EV-52 (5 mg/kg) *n* = 6, Npc control *n* = 6, and Npc UB-EV-52 (5 mg/kg) = 6). * *p* < 0.05; ** *p* < 0.01; *** *p* < 0.001.

As expected, mutant mice exhibited a highly inflammatory profile with increases in several proinflammatory cytokines, such as *Il-1β and Tnf-α,* compared to WT mice (Figure 4A,B). In addition, we reviewed other neuroinflammatory markers, such as *monocyte chemoattractant protein 1* (*Mcp1*) and the brain tissue astroglial marker *glial fibrillar acidic protein* (*Gfap*), which are significantly increased in Npc mice (Figure 4C,D). Likewise, a significant reduction in *Il-1β* and *Mcp1* among UB-EV-52-treated Npc mice compared with untreated littermates was determined (Figure 4A–C). Finally, a clear tendency to decrease *Tnf-α* gene expression was observed in UB-EV-52-treated Npc mice compared with untreated littermates. Although it did not reach significance, it is worth noting the degree of reduction in Wt levels (Figure 4B).

To address the oxidative scenario in Npc mice, we evaluated the gene expression of *heme oxygenase decycling 1* (*Hmox1*) and *inducible nitric oxidase* (*iNOS*). Both enzymes are associated with OS status. We found a decrease in *Hmox1* gene expression in Npc mice compared to Wt, which was reversed under UB-EV-52 treatment. Likewise, *iNOS* levels decreased in the Npc group compared with the Wt group and were partially reversed in Npc-treated animals (Figure 4E,F).

### 2.6. Decrease of Autophagic Markers and Increased Synaptic Markers Promoted by UB-EV-52 Treatment in Npc Mice.

**Figure 5:**
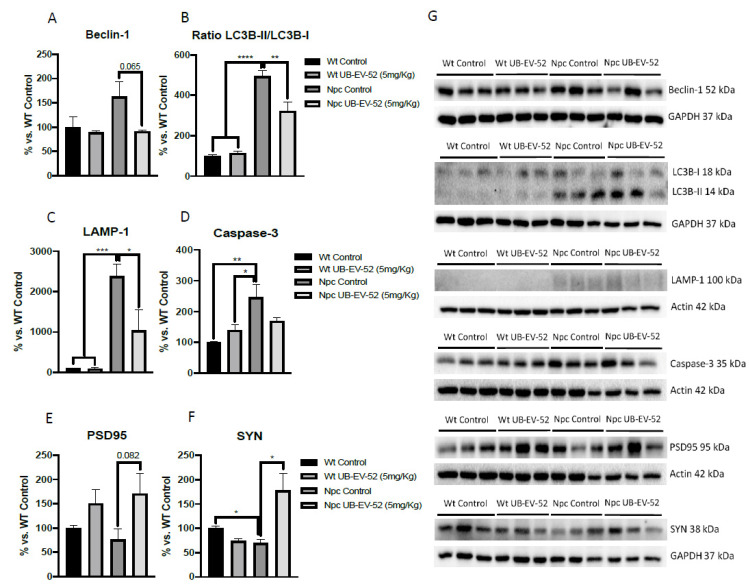
Quantifications and representative Western blot for beclin-1 (**A**–**G**), ratio LC3B-II/LC3B-I (**B**–**G**), LAMP-1 (**C**–**G**), caspase-3 (**D**–**G**), PSD95 (**E**–**G**) and SYN (**F**,**G**) from hippocampal tissue in females and males. Bar graphs values are 100% adjusted for Wt control protein levels. Values are mean ± standard error of the mean (SEM); (*n* = 6 for each group). * *p* < 0.05; ** *p* < 0.01; *** *p* < 0.001; **** *p* < 0.0001.

The autophagic process was studied by beclin-1 protein levels, microtubule-associated proteins 1A/1B light chain 3B (LC3B) and lysosome-associated membrane glycoprotein 1 (LAMP-1). The results showed that Npc mice had higher beclin-1, LCB-II/I ratio and LAMP-1 protein levels than their Wt littermates (Figure 5A–C,G). In addition, when mice were under treatment with UB-EV-52, a decrease in beclin-1, LCB-II/I ratio and LAMP-1 were determined. Overall, these results indicated a decrease in the autophagic process in sEHi treated mice. In addition, like the intended change in autophagy, EV-UB-52 reduced caspase-3 protein expression in Npc mice, which is upregulated compared with the Wt group of mice (Figure 5D,G).

The neuroprotective effects of sEH inhibition were determined by measuring synaptic markers, such as synaptophysin (SYN) and postsynaptic density 95 (PSD95). In both cases, Npc mice showed reduced levels compared with the Wt group (Figure 5E–G). As expected, UB-EV-52 treatment increased the protein content of SYN and PSD95 in Npc mice (Figure 5E–G).

## 3. Discussion

Despite various therapeutic approaches, only modest clinical improvements have been achieved for treating NPC disease. Consequently, its treatment is an unmet clinical need, being necessary to explore new therapeutic strategies [8]. This study focuses on establishing a relationship between cognitive improvement, anti-inflammatory and antioxidant effects and the reduction of the autophagic process in the brain of the NPC mouse model after treatment with UB-EV-52, a well-known, potent and specific sEHi [21]. We evaluated the impact of pharmacological inhibition of sEH on NPC phenotypic characteristics, such as lipid accumulation, cognitive impairment and other health parameters, such as weight gain/loss and survival. Inhibition of the sEH pathway led to an increase in EETs, leading to an anti-inflammatory action that triggered a cascade of molecular and cellular events, such as modulation of OS, mitochondrial function, ER stress or autophagic process [32,33].

Of paramount importance, oral treatment with UB-EV-52 increased the lifespan of Npc mice by 25% and improved weight gain, and reduced symptoms of tremor and unstable gait that were visually detectable in Npc mice at postnatal week seven without sex bias. Moreover, motor behavior and cognition differences were determined at earlier stages (fifth to the eighth week) [31]. Accordingly, we observed those changes described above as earlier as weaning time (21 postnatal days), and OF and EPM tests corroborated them. In addition, treatment with sEHi improved locomotor activity, as well as reduced some anxiety-like readouts, such as rearings, or the time that mice spend in open arms in the EPM test. Cognitive impairment is also a severe symptom in the NPC pathology that was reverted after treatment with sEHi. Treatment with UB-EV-52 prevented the cognitive impairment characteristic of the Npc mouse model, demonstrated by the high DI obtained in the NORT test, both in the short- and long-term, without sex bias. Because EV-UB-52 treatment successfully rescued behavioral and cognitive impairment in the Npc mouse model, we focused on the cellular and molecular mechanisms involved in the positive action induced by sEHi.

Many neurodegenerative diseases share inflammatory and atypical OS processes as two main pathological events [34]. Therefore, the particular insight of this study was to demonstrate that the Npc mice model had a significant increase in gene expression for inflammatory markers, such as *Il-1β*, *Tnf-α*, that was prevented after sEHi treatment (Figure 4A,B). Inflammation spread to brain tissue inducing a significant increase in *Mcp1* and *Gfap*, reflecting the presence of astrogliosis in Npc mice that was also rescued after UB-EV-52 treatment (Figure 4C,D). In summary, these results reinforce the hypothesis that increased endogenous anti-inflammatory EETs reduced disease progression in the Npc mouse model. In contrast, when we checked the OS markers *Hmox1* and *iNOS*, although slight changes were determined in the Npc mice compared to the Wt group, they were not statistically significant. However, OS parameters were partially modified by treatment with sEHi. These results indicate that in the Npc mouse model used, OS does not play a key role in the pathogenesis of the disease. In any case, there is evidence that OS is a player present in humans and in most animal models of this disease, and the production of reactive oxygen species was prevented by α-Tocopherol [35,36]. Therefore, it is plausible that sEHi recovered the homeostasis of cellular processes, positively impacting OS parameters after UB-EV-52 in Npc mouse model. Notably, UB-EV-52 and other sEHi have also shown beneficial effects in different mouse models for neurodegenerative or metabolic diseases [21,33].

The pathological processes leading to neurodegeneration in many lysosomal storage disorders are due to an imbalance between induction and inhibition of autophagy [37,38]. Specifically, NPC1 deficiency is characterized by increasing autophagic process inducing increases accumulation of ubiquitinated proteins in the mutant mouse brain [15]. Thus, several authors have reported similar findings confirming the induction of autophagy in brain, liver or primary human fibroblasts from NPC patients. Induction of autophagy and increased beclin-1 levels is similarly observed in primary human fibroblasts deficient in NPC2 and a chemical model inducing accumulation of unesterified cholesterol by U18666A [39]. Accordingly, we found an increase in beclin-1 protein levels in Npc mice model, as well as inhibition of sEH by UB-EV-52, which was able to reduce beclin-1 protein levels. These results indicated that although no remarkable changes in lipid content occurred, inhibition of sEH promoted the reduction of autophagy in the neuronal tissue of the murine Npc model.

Furthermore, several studies have shown that LC3 levels are not modified by inhibition of lysosome function in pathological situations characterized by altered autophagosome-lysosome fusion, but rather the ratio between LC3B-I and -II forms changes [40]. Consistent with this point, we found an increase in the LC3B-II form compared to LC3B-I, thus increasing the II/I ratio, which demonstrated activation of autophagy. Npc mice treated with UB-EV-52 reversed the II/I ratio, indicating a reversal in the autophagic process, which positively impacted disease progression, as demonstrated by the phenotypic results presented above (Figure 5B).

To further study autophagy abnormalities in the Npc mice model used and the impact of sEHi treatment, we determined the levels of LAMP1 protein. LAMP1 is a lysosomal protein involved in the completion of the macroautophagy process through the formation of autophagolysosome, allowing the initiation of lysosomal activity to degrade proteins, among others [41,42]. As for NPC, LAMP1 is associated with cholesterol trafficking into cells and the lysosome and is, therefore, related to the etiopathology of NPC. Overexpression of LAMP1 in HeLa cells rescued U18666A-induced cholesterol accumulation and reduced LAMP1 levels based on the beneficial pharmacological action of cyclodextrin [39]. Recent studies demonstrated a highly glycosylated form of LAMP1 in the NPC1 mice model that correlated neuronal loss [43]. In Npc mice, a significant increase in LAMP1 protein levels was found, in agreement with the observed changes in the ratio of beclin-1 and LC3B, thus signaling the termination of the autophagic process in this model. Notably, treatment with sEHi strongly reduced LAMP1 and caspase-3 protein levels, supporting the positive pharmacological effect of UB-EV-52 on the autophagy and apoptotic signaling pathway in these Npc mice model (Figure 5C,D). Although, in our hands, cholesterol levels are not drastically changed after UB-EV-52 treatment, a slight effect was observed. Therefore, the effect of sEHi on LAMP1-mediated cholesterol trafficking to the lysosome cannot be ruled out and may be considered a secondary mechanism to explain the beneficial effects of increasing levels of EETs by sEH inhibition.

Finally, another characteristic feature of NPC disease is abnormal synaptic plasticity, promoting memory impairment and dementia [44]. Here, we found reduced levels in synaptic markers between the Npc control group and the Wt group, being significant for SYN. Moreover, significant changes in the synaptic marker SYN and a clear trend for PSD95 in the brain between Npc-treated mice groups and Npc controls demonstrate neuroprotective effects under sEH inhibition treatment. Consistent with these results, several studies have described changes in the levels of synaptic proteins, such as syntaxin-1A, amphiphysin, complexin-1, SYN, PSD95, among others, in lysosomal storage diseases, such as NPC disease [45]. In addition, these observations regarding sEHi treatment are in agreement with previous reports that demonstrated that long-term administration of TPPU, a well-characterized sEHi, to the 5xFAD mouse model of AD also rescued SYN and PSD95 levels [46], suggesting that the improvement of synaptic plasticity and cognitive performance in the NPC mice model could be attributed to sEH inhibition.

## 4. Materials and Methods

### 4.1. Animals

The animals used were generated by Gómez-Grau et al. [31]; in brief, the heterozygous *Npc1^imagine/+^* and *Npc1^pioneer/+^* mice have a C57BL/6 genetic background and were kindly provided to us for this study by the Addi and Cassi Fund (http://addiandcassi.com/) after generation by the Ozgene company. The heterozygous *Npc1^imagine/+^* mice were interbred and generated litters composed of *Npc1^imagine/imagine^*, *Npc1^imagine/+^* and *Npc1^+/+^* mice. Heterozygous *Npc1^pioneer/+^* mice were interbred and generated litters consisting of *Npc1^pioneer/pioneer^*, *Npc1^pioneer/+^* and *Npc1^+/+^* mice. Homozygotes for the imagine and pioneer mutation, and hereafter we refer to mutant animals as Npc mice to simplify readout, while we used non-mutant littermates as Wt controls. Genotyping analysis was performed as previously described by Gómez-Grau et al. [31].

Animals had free access to food and water and were maintained under standard temperature conditions (22 ± 2 °C) and 12 h: 12 h light–dark cycles (300 lux/0 lux).

Wt and Npc mice (*n* = 48) were used to perform the cognitive tests followed by molecular analysis. Animals were randomly divided into four groups: Wt group (*n* = 12; females *n* = 6; males *n* = 6), NpcC group (*n* = 12; females *n* = 6; males *n* = 6), UB-EV-52-treated Wt group (Wt UB-EV-52) (*n* = 12; females *n* = 6; males *n* = 6), and Npc group treated with UB-EV-52 (Npc UB-EV-52) (*n* = 12; females *n* = 6; males *n* = 6). For the survival experiment, Npc mice (*n* = 24; females *n* = 12; males *n* = 12) were randomly divided into two groups: Npc group (*n* = 12; females *n* = 6; males *n* = 6), and NPC group treated with UB-EV-19 (Npc UB-EV-52) (*n* = 12; females *n* = 6; males *n* = 6).

UB-EV-52 was dissolved in 2% polyethylene glycol 400 (PEG400) at 5 mg/kg/day and administered through drinking water from weaning (1-month-old). Control groups also received the vehicle in the drinking water. After four weeks of treatment, behavioral tests were performed on the animals, and the drug was administered up to sacrifice. The UB-EV-52 was administered in the drinking water from weaning (1-month-old) until natural death. Water consumption was monitored weekly, and concentrations were adjusted accordingly to achieve the optimal dose for each cage.

The studies were conducted in accordance with the Institutional Guidelines for the Care and Use of Laboratory Animals established by (European Communities Council Directive 2010/63/EU and Guidelines for the Care and Use of Mammals in Neuroscience and Behavioral Research, National Research Council 2003) and were and were approved by the Institutional Animal Care and Use Committee of the University of Barcelona (670/14/8102, approved at 14 November 2014) and by Generalitat de Catalunya, Spain (10291, approved at 28 January 2018). Every effort was made to reduce the number of animals and their suffering.

### 4.2. Cognitive Tests

#### 4.2.1. Open Field

The open-field test (OFT) is an experiment used to assess general locomotor activity and anxiety in rodents. The test is based on the rodent’s fear and anxiety of being in bright, open spaces. The OFT apparatus was a white wooden box (50 cm × 50 cm × 25 cm). The arena was divided into two areas defined as central and peripheral zone (15 cm between the central zone and the wall). Trials were recorded for later analysis using a camera placed on top of the apparatus, and changes in movements were scored using SMART^®^ ver.3.0 software. Each trial began by placing the mice in the center and exploring the box for 5 min. The trial ended by returning the mice to the cages. Among trials, the OFT apparatus was carefully cleaned (with a cloth soaked in 70% ethanol). The parameters scored were the duration of time spent in the center, rearings, and distance traveled, calculated as the total distance traveled in 5 min.

#### 4.2.2. Elevated Plus Maze

The elevated plus maze (EPM) test is based on the preference of mice for closed and dark places, thus assessing anxiety-related behaviors and risk-taking. The EPM apparatus consists of two open and two closed arms elevated 50 cm above the ground. Mice were placed on the central platform, facing the open arms, and allowed to explore the apparatus for 5 min. Afterward, the mice were returned to their cages, and the arms of the EPM were cleaned with 70% ethanol to avoid any olfactory clues. Behavior was scored using SMART^®^ ver. 3.0 software (Panlab, Cornellà, Barcelona, Spain), and each trial were recorded for subsequent analysis. Parameters evaluated included time spent in the open and closed arms, rearings, defecation and urination.

#### 4.2.3. Novel Object Recognition Test

The novel object recognition test (NORT) is a cognitive test used to assess short- and long-term recognition memory. The apparatus used consisted of a black polyvinyl chloride 90º black maze, two-arm, 25 cm long, 20 cm high and 5 cm wide. The objects to be discriminated against were made of plastic and were chosen so as not to frighten mice without any parts susceptible to being bitten. The test was performed for 5 days, and on the first three days, the animals were individually habituated to the apparatus for 10 min. On the fourth day, the animals were individually exposed for 10 min to the apparatus and freely explored the area inside the apparatus (First trial/Familiarization), where we placed two identical novel objects (A + A’ or B + B’) at the end of each arm. After 10 min, the animals were removed and returned to their cage, and 2 h later, the retention trial (second trial) was performed. In the second trial, objects A’ and B’ were exchanged (A + B’ or A’ + B), and the mice were allowed to explore the maze for 10 min. Twenty-four hours later the first trial, the animals were again exposed to the apparatus, and in this case, objects A’ and B’ were replaced by two new objects with different shapes and colors (A + C or B + C’), and they were allowed to explore them for 10 min. The exploration time of the new object (TN) and the old object (TO) was measured with a camera, and videos were recorded. Object exploration was defined as pointing the nose towards the object at a distance ≤ of 2 cm and/or touching it with the nose. Turning or sitting around the object was not considered exploration. To avoid object preference biases, objects A and B were counterbalanced so that half of the animals in each experimental group were exposed first to object A and then to object B, while the other half saw object B first and then object A. Finally, to obtain cognitive performance, the discrimination index (DI), defined as (TN-TO)/(TN + TO), was calculated.

#### 4.2.4. Object Location Test (OLT)

The object location test (OLT) is a well-established task based on the spontaneous tendency of rodents to spend more time exploring the location of a novel object than that of a known object, as well as to recognize when an object has been relocated. The test was conducted over 3 days in a wooden box (50 cm × 50 cm × 25 cm), in which three walls were white except for one, which was black. On the first day, the box was empty, and the animals were only habituated to the sand in the open field for 10 min. On the second day, two objects were placed in front of the black wall, equidistant from the wall. The objects were 10 cm high and identical. The animals were placed in the open field arena and allowed to explore both the objects and the environment for 10 min. Afterward, the animals were returned to their cages, and the OLT apparatus was cleaned with 70% ethanol. On the third day, an object was moved in front of the white wall to test spatial memory. Trials were recorded with a camera mounted over the open field area, and total exploration time was determined by scoring the amount of time (seconds) spent sniffing the object in the new location (TN) and the object in the old location (TO). To assess cognitive performance, DI was calculated, which is defined as (TN-TO)/(TN + TO).

### 4.3. Tissues Preparation

After three days of cognitive and memory testing, all groups of mice were euthanized, tissues (liver and brain) were isolated and frozen in powdered dry ice and stored at −80 °C for later use.

### 4.4. Protein Level Determination by Western Blotting

For protein extraction, tissue samples were homogenized in lysis buffer containing phosphatase and protease inhibitors (cocktail II, Sigma-Aldrich, St. Louis, MO, USA). Total protein levels were obtained, and protein concentration was determined by the Bradford method. Fifteen μg of protein samples were separated by sodium dodecyl sulfate-polyacrylamide gel electrophoresis (SDS-PAGE) (8–20%) and transferred to polyvinylidene difluoride (PVDF) membranes (Millipore). The membranes were then blocked in 5% nonfat milk in Tris-buffered saline (TBS) containing 0.1% Tween-20 TBS (TBS-T) for 1 h at room temperature, followed by overnight incubation at a 4 °C with the primary antibodies listed in Table S3. The membranes were then washed and incubated with secondary antibodies for 1 h at room temperature. Immunoreactive proteins were visualized with the chemiluminescence-based detection kit, following the manufacturer’s protocol (ECL Kit; Millipore, Billerica, MA, USA), and digital images were acquired using ChemiDoc XRS+ System (BioRad, Hercules, CA, USA). Semiquantitative analyses were performed using ImageLab software (BioRad, Hercules, CA, USA), and results were expressed in Arbitrary Units (AU), considering control protein levels as 100%. Protein load was routinely monitored by immunodetection of glyceraldehyde-3-phosphate dehydrogenase (GAPDH).

### 4.5. RNA Extraction and Gene Expression Determination

Isolation of total RNA from tissue samples was performed with TRIsure™ reagent following the manufacturer’s instructions (Bioline Reagent, London, UK). RNA yield, purity, and quality were determined spectrophotometrically with a NanoDrop™ ND-1000 (Thermo Scientific) apparatus and an Agilent 2100B Bioanalyzer (Agilent Technologies). RNAs with 260/280 and RIN ratios greater than 7.5, respectively, were selected. Reverse transcription-polymerase chain reaction (RT-PCR) was performed as follows: 2 μg of messenger RNA (mRNA) was transcribed using the high-capacity cDNA reverse transcription kit (Applied Biosystems, Foster City, CA, USA). Quantitative real-time PCR (qPCR) was used to quantify mRNA expression of the inflammatory and OS marker genes listed in Table S4.

SYBR^®^ Green real-time PCR was performed on a Step One Plus detection system (Applied Biosystems, Foster City, CA, USA)employing SYBR^®^ Green PCR Master Mix (Applied-Biosystems). Each reaction mixture contained 6.75 μL of complementary DNA (cDNA) (which concentration was 2 μg), 0.75 μL of each primer (which concentration was 100 nM), and 6.75 μL of SYBR^®^ Green PCR Master Mix (2X).

Data were analyzed utilizing the comparative cycle threshold (Ct) method (ΔΔCt), in which the housekeeping gene level was used to normalize for differences in loading and sample preparation. Normalization of expression levels was performed with *β-actin* for SYBR^®^ Green-based real-time PCR results. Each sample was analyzed in duplicate, and the results represent the n-fold difference in transcript levels between the different groups.

### 4.6. Cholesterol Measurement

Liver and brain tissue was homogenized using TissueRuptor (QIAGEN, Hilden, Germany) in PBS at 5% *w/v.* Lipids were extracted with a chloroform:methanol (2:1) mixture containing stigmasterol as an internal standard. Samples were derivatized with N,O-bis(trimethylsilyl) trifluoroacetamide (BSTFA) to form trimethylsilyl derivatives and analyzed by gas chromatography-mass spectrometry. Gas chromatography coupled to electron impact (70 eV) mass spectrometry was carried out using a Fisons gas chromatograph (8000 series) coupled to a Fisons MD-800 mass selective detector. The system was equipped with an HP-5-MS capillary column (30 m × 0.20 mm inner diameter), which was programmed to increase from 120 °C to 315 °C, at 5 °C/minute after an initial delay of 2 min. Analyses were performed in the selected ion monitoring mode. The ions selected were those at *m*/*z* 129, 458 and 484.

### 4.7. Sphingolipid Determination

Homogenized liver and brain tissue was homogenized using TissueRuptor (QIAGEN) in 0.2 M sucrose at 5% *w/v*. Protein concentration was determined by the Lowry method, and a 400 μg protein aliquot was used for sphingolipid quantification. Sphingolipid extracts, enriched with internal standards, were prepared, and different lipid groups, including ceramides, sphingomyelins, and glycosphingolipids, were analyzed by liquid chromatography-mass spectrometry (LC/MS). The LC/MS analysis consisted of a Waters Acquity UPLC system connected to a Waters LCT Premier accelerated time-of-flight orthogonal mass spectrometer (Waters), operated in positive and negative electrospray ionization modes. Full scan spectra were acquired from 50 to 1500 Da, and individual spectra were summed to produce data points every 0.2 s. Mass accuracy and reproducibility were maintained using an independent reference spray via the LockSpray interference. The analytical column was a 1.7-μm ethylene-bridged hybrid (C8 Acquity UPLC column, Waters), measuring 100 mm × 2.1 mm (inner diameter). The two mobile phases were MeOH:HCOOH (998:2, *v*/*v*) for phase A and water:HCOOH (998:2, *v/v*) for phase B, both with 2 mM ammonium formate. The column was kept at 30 °C. Quantification was performed from the chromatogram of extracted ions of each compound, using 50 MDA windows. The linear dynamic range was determined by injecting standard mixtures. Positive identification of compounds was based on accurate mass measurement with an error of <5 ppm and their retention time in the LC compared with that of a standard (±2%).

### 4.8. Data Acquisition and Statistical Analysis

Data analysis was performed with GraphPad Prism ver. 8 statistical software. Data are expressed as mean ± standard error of the mean (SEM) of at least 3 samples per group. Strain and treatment effects were compared by two-way analysis of variance (ANOVA), followed by Tukey’s post hoc analysis or two-tail Student’s t-test when necessary. Statistical significance was considered to be present when *p*-values were <0.05. Statistical outliers were determined with Grubbs’ test and, when necessary, removed from the analysis. The cognitive analysis was performed blind. The person who evaluated the videos was different from the person who performed the cognitive tests. In addition, the videos were named with a blind code to avoid bias in the analysis.

## 5. Conclusions

Our results reinforce sEH inhibition as a promising therapeutic approach for Npc disease. Thus, we have demonstrated a positive rescue of the Npc mouse model phenotype, improving survival and motor activity, as well as cognitive outcomes. As for the biochemical and molecular alterations determined, these were modified by inhibition of sEH using a potent and specific inhibitor, UB-EV-52, permeable to the blood–brain barrier (BBB) and orally available. In addition to the anti-inflammatory effect observed after treatment with sEHi, changes in OS were demonstrated, along with modulation of autophagy, modifications in lipid storage, and synaptic plasticity enhancement. In the future, further studies are needed to unravel the involvement of sEH in the observed beneficial effects on phenotype and cognition.

## Data Availability

Not applicable.

## Supplementary Materials

The following are available online at https://www.mdpi.com/article/10.3390/ijms22073409/s1.
