# Supplementary material for: Inhibition of Soluble Epoxide Hydrolase Ameliorates Phenotype and Cognitive Abilities in a Murine Model of Niemann Pick Type C Disease
Source: Int J Mol Sci. 2021 Mar 26;22(7):3409. doi: 10.3390/ijms22073409 (PMC8036710; doi:10.3390/ijms22073409)
Supplement: Supplementary file 1 [file ijms-22-03409-s001.pdf]

Supplementary Figure S1

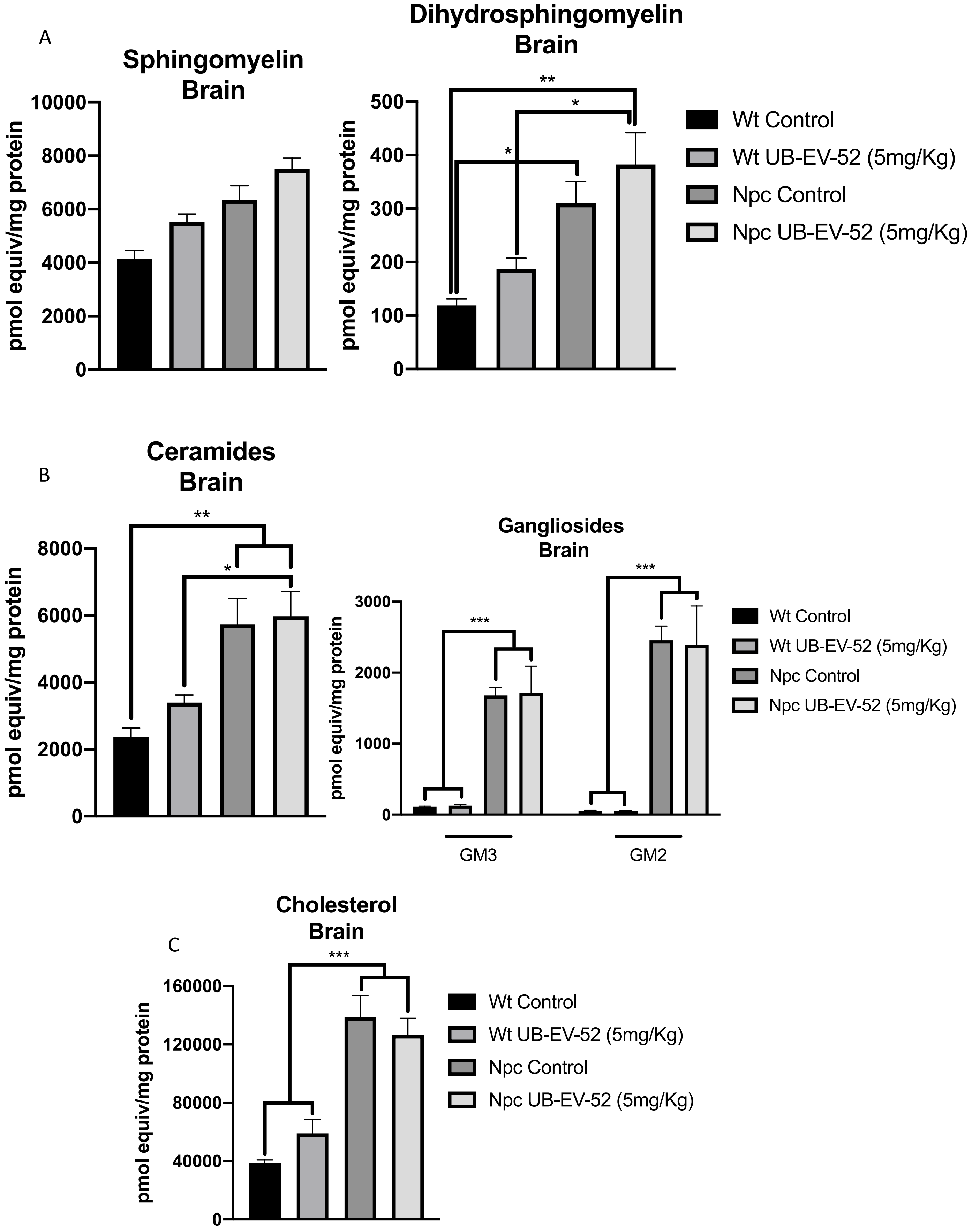

Supplementary Figure S2

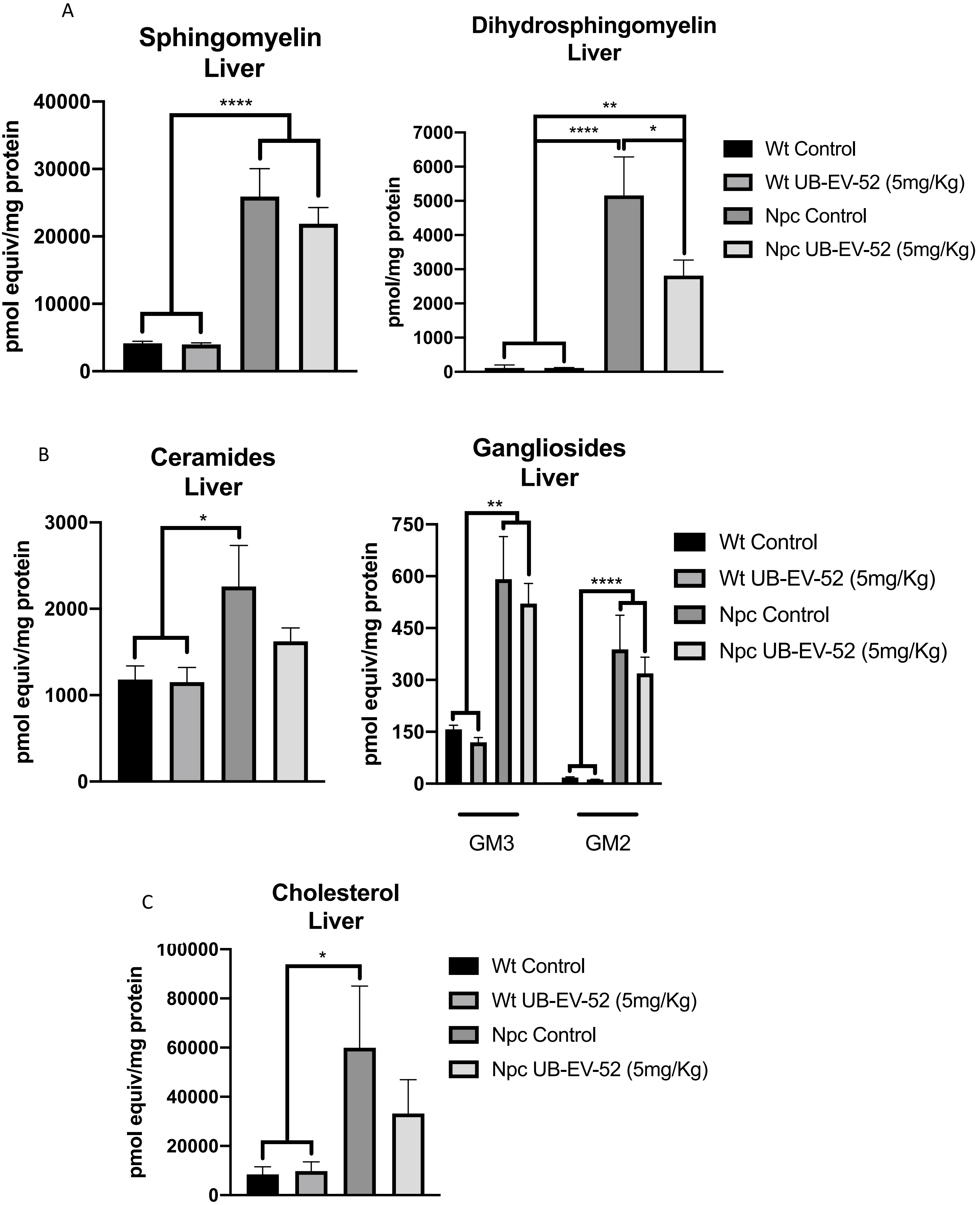

Table S1. Parameters measured in the Open Field Test (OFT). (n): number of events. Results are expressed as a mean ± Standard error of the mean (SEM). **p <0.01; ****p <0.0001 vs Wt Control. #p <0.05 vs Npc Control.

	Wt Control	Wt UB-EV-52 (5mg/Kg)	Npc Control	Npc UB-EV-52 (5mg/Kg)
**Locomotor activity (cm)**	3,447.65 ± 248.64	3,584.27 ± 219.79	1,359.70 ± 121.98****	1,832.89 ± 152.47$^{****,\#}$
**Distance in zone-Center (cm)**	65.72 ± 10.01	76.73 ± 10.70	106.64 ± 1.53****	93.78 ± 8.75$^{**,\#}$
**Distance in zone-Periphery (com)**	3,148.03 ± 193.09	3,298.81 ± 162.98	1,410.80 ± 120.00****	1,802.90 ± 135.98$^{****,\#}$
**Rearings (n)**	36.30 ± 6.68	43.80 ± 2.97	17.25 ± 5.20	16.38 ± 2.65
**Grommings (n)**	2.30 ± 0.52	3.80 ± 0.42	0.75 ± 0.31****	1.63 ± 0.26****
**Defecations (n)**	0.70 ± 0.26	0.50 ± 0.22	0.38 ± 0.18	0.50 ± 0.27
**Urinations (n)**	0.30 ± 0.15	0.50 ± 0.22	0.25 ± 0.16	0.38 ± 0.18

Table S2. Parameters measured in the Elevated Plus Maze Test (EPM). (n): number of events. Results are expressed as a mean ± Standard error of the mean (SEM). **p <0.01; ***p<0.001; ****p <0.0001 vs Wt Control. #p <0.05; ##p <0.01 vs Npc Control.

	Wt Control	Wt UB-EV-52 (5mg/Kg)	Npc Control	Npc UB-EV-52 (5mg/Kg)
**Total Distance (cm)**	1,492.94 ± 85.63	1,386.85 ± 66.07	896.92 ± 102.80***	1,057.12 ± 96.57**
**Time in Center Zone (sec)**	3.87 ± 0.38	4.25 ± 0.41	17.72 ± 1.85	14.12 ± 1.07
**Time in Open Arms (sec)**	10.74 ± 1.68	11.25 ± 0.87	8.39 ± 1.05	11.65 ± 5.18
**Time in Closed Arms (sec)**	85.39 ± 1.88	84.50 ± 0.98	73.87 ± 2.73	73.30 ± 6.16
**Rearings (n)**	54.10 ± 3.72	44.90 ± 2.54	8.50 ± 0.91****	19.88 ± 2.97$^{****,\#\#}$
**Defecations (n)**	0.30 ± 0.15	0.60 ± 0.22	0.88 ± 0.30	0.88 ± 0.48
**Urinations (n)**	0.30 ± 0.15	0.00 ± 0.00*	0.25 ± 0.16	0.00 ± 0.00$^{*,\#}$

Table S3. Antibodies used in Western blot studies.

Antibody	Host	Source/Catalog	WB dilution
**Beclin-1**	Rabbit	Cell signaling/#3738	1:1000
**SYN**	Rabbit	Dako/CloneSY38	1:2000
**PSD95**	Rabbit	Abcam/ab18258	1:1000
**LC3B**	Rabbit	Cell signaling/#2775S	1:1000
**LAMP-1**	Mouse	Santa Cruz/sc-19992	1:1000
**Caspase-3**	Rabbit	BD Transduction Laboratories/C31720	1:1000
**Actin**	Mouse	Sigma-Aldrich/A5441	1:2000
**GAPDH**	Mouse	Millipore/MAB374	1:5000
**Goat-anti-mouse HRP conjugated**		Biorad/170-5047	1:2000
**Goat-anti-rabbit HRP conjugated**		Biorad/170-6515	1:2000

Table 4. Primers and probes used in qPCR studies.

SYBR Green primers

Target	Product size (bp)	Forward primer (5'-3')	Reverse primer (5'-3')
Hmox1	177	TGACACCTGAGGTCAAGCAC	GTCTCTGCAGGGGCAGTATC
Tnf-α	157	TCGGGGTGATCGGTCCCCAA	TGGTTTGCTACGACGTGGGCT
Il-1β	179	ACAGAATATCAACCAACAAGTGATATTCTC	GATTCTTTCCTTTGAGGCCCA
Mcp1	159	CCCACTCACCTGCTGCTACT	TCTGGACCCATTCCTTCTTG
iNOS	189	GGCAGCCTGTGAGACCTTTG	GAAGCGTTTCGGGATCTGAA
Gfap	125	CCTTCTGACACGGATTTGGT	ACATCGAGATCGCCACCTAC
β-actin	190	CAACGAGCGGTTCCGAT	GCCACAGGTTCCATACCCA
